# 3,9′-Bi(9*H*-fluorene)

**DOI:** 10.1107/S1600536812024841

**Published:** 2012-06-13

**Authors:** Jie Liu, Wentao Yu

**Affiliations:** a State Key Laboratory of Crystal Materials, Shandong University, Shanda Nanlu 27 Jinan 250100, People’s Republic of China

## Abstract

The title compound [systematic name: 9-(9*H*-fluoren-3-yl)-9*H*-fluorene], C_26_H_18_, was obtained unintentionally as the product of the synthesis of a compound based on fluorene–thio­phene units. The two fluorene rings are connected through C atoms in the 3- and 9′-positions, and the dihedral angle between the mean planes of the two fluorene units is 78.57 (6)°.

## Related literature

For the crystal structures of related compounds, see: Dougherty *et al.* (1978 ▶); Sridevi *et al.* (2006 ▶). For the synthesis of the compound, see: Stille *et al.* (1993 ▶, 1998 ▶); Grasa & Nolan (2001 ▶). For the inter­molecular C—H⋯π inter­actions, see: Tsuzuki *et al.* (2000 ▶); Nishio (2004 ▶).
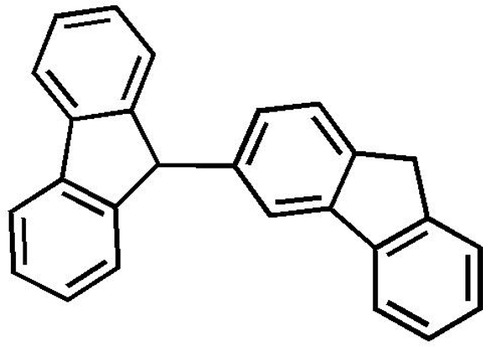


## Experimental

### 

#### Crystal data

C_26_H_18_
*M* *_r_* = 330.40
Orthorhombic, 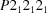

*a* = 6.22600 (1) Å
*b* = 8.3968 (2) Å
*c* = 33.5357 (7) Å
*V* = 1753.20 (6) Å^3^
*Z* = 4
Mo *K*α radiation
μ = 0.07 mm^−1^
*T* = 293 K
0.45 × 0.22 × 0.16 mm

#### Data collection

Bruker APEXII CCD area-detector diffractometer
Absorption correction: multi-scan (*APEX2*; Bruker, 2005 ▶) *T*_min_ = 0.969, *T*_max_ = 0.989
15454 measured reflections
2352 independent reflections
2014 reflections with *I* > 2σ(*I*)
*R*_int_ = 0.032

#### Refinement

*R*[*F* ^2^ > 2σ(*F* ^2^)] = 0.035
*wR*(*F* ^2^) = 0.091
*S* = 1.04
2352 reflections
235 parameters
H-atom parameters constrained
Δρ_max_ = 0.11 e Å^−3^
Δρ_min_ = −0.14 e Å^−3^

### 

Data collection: *APEX2* (Bruker, 2005 ▶); cell refinement: *APEX2*; data reduction: *APEX2*; program(s) used to solve structure: *SIR97* (Altomare *et al.*, 1999 ▶); program(s) used to refine structure: *SHELXL97* (Sheldrick, 2008 ▶); molecular graphics: *SHELXTL* (Sheldrick, 2008 ▶); software used to prepare material for publication: *WinGX* (Farrugia, 1999 ▶) and *Mercury* (Macrae *et al.*, 2006 ▶).

## Supplementary Material

Crystal structure: contains datablock(s) I, global. DOI: 10.1107/S1600536812024841/zj2080sup1.cif

Structure factors: contains datablock(s) I. DOI: 10.1107/S1600536812024841/zj2080Isup2.hkl

Supplementary material file. DOI: 10.1107/S1600536812024841/zj2080Isup3.mol

Supplementary material file. DOI: 10.1107/S1600536812024841/zj2080Isup4.cml

Additional supplementary materials:  crystallographic information; 3D view; checkCIF report

## Figures and Tables

**Fig. 1.**
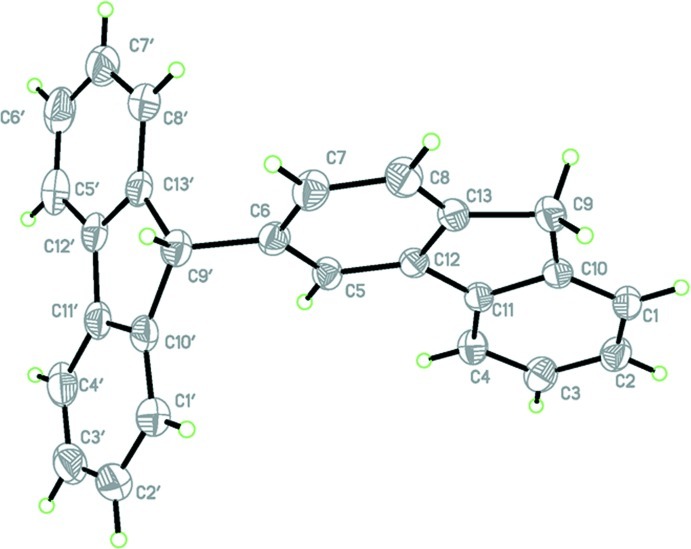
The molecular structure of the title compound with 30% probability displacement ellipsoids.

**Fig. 2.**
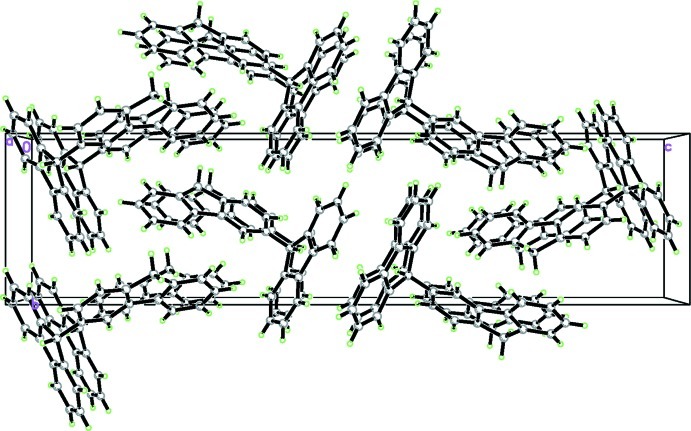
Part of the packing of the title compound, viewed down the a direction.

**Fig. 3.**
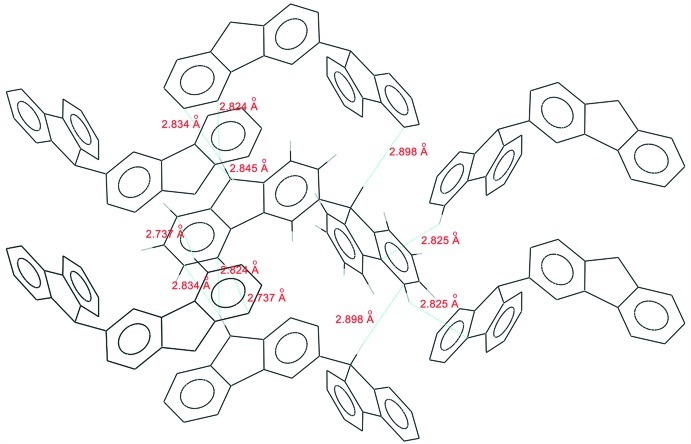
A view of the C—H ··· π interactions (dotted lines) in the crystal structure of the title compound.

**Fig. 4.**
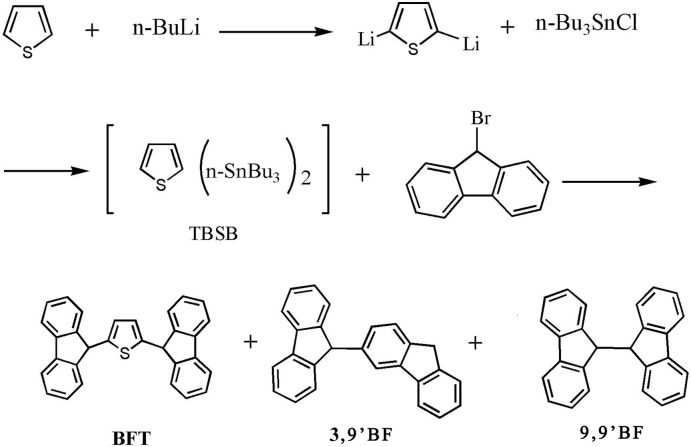
Reaction scheme showing the formation of 3,9'-BF, 9,9'-BF and 2,5-bis(9H-fluoren-9-yl)thiophene (BFT).

## supplementary crystallographic information

### Comment

The molecule of the title complound (I) (Fig. 1) as the isomer of 9,9'-bi-9*H*-fluorene (9,9'-BF) is noncentrosymmetric, and the space group is *P*2_1_2_1_2_1_. The two fluorene groups of the compound are like the letter 'T' in shape with a dihedral angle of 78.57 (6)°. Also, it is found that benzene rings of the fluorene units are not in the same plane, and the dihedral angles are 10.54 (6) and 5.84 (6)°, respectively. The crystal packing is stabilized by intermolecular C—-H···π interactions (Fig. 3).

### Experimental

The title compound, 3,9'-BF, was obtained unintentionally as the product of an attempted synthesis of 2,5-bis(9*H*-fluoren-9-yl)thiophene through Still reaction method. *n*-Butyllithium (20 ml, 2.5 *M* in hexane, 50 mmol) was added dropwise at -78 °C into a consistently stirred mixture of thiophene (22 mmol, 1.8 ml) and dry THF (80 ml), and the mixture would be with further stirring for 2 h at room temperature under an atmosphere of dry argon. After cooling the reaction mixture to -78 °C tri-*n*-butyltin chloride (15 ml) was added drop-wise to the mixture system. Then, the mixture was stirred continuously over one night before being poured into saturated NH_4_Cl water solution (100 ml). After extraction with diethyl ether, the organic layer was dried over anhydrous MgSO_4_ and the yellow fluid bis[tri-*n*-butyltin] thiophene (TBSB) was obtained. Furthermore, DMF (10 ml) was added to the mixture of TBSB (2.5 mmol, 1.654 g), 9-bromo-fluorene (6.25 mmol, 1.53 g) and potassium fluoride (2.5 mmol, 0.145 g) with stirring about 15 min. Appropriate amount of tetrakis (triphenylphosphine) palladium (0) was added to the stirring system and refluxed at 100 °C for 16 h under an atmosphere of dry argon. After extraction with dichloromethane (30 ml), the mixture was purified by silica-gel column chromatography to give 3,9'-BF, 9,9'-BF and 2,5-bis(9*H*-fluoren-9-yl)thiophene. Finally, single crystals of 3,9'-BF were obtained by recrystallizing from dichloromethane.

### Refinement

All the H atoms were positioned geometrically [C–H = 0.93, 0.96 and 0.98 Å] and refined using a riding model with *U*iso (H) = 1.2 *U*eq (C). In the absence of significant anomalous scattering, Friedel pairs were merged; the absolute configuration was not determined.

### Crystal data

**Table:**

C_26_H_18_	*F*(000) = 696
*M**_r_* = 330.40	*D*_x_ = 1.252 Mg m^−^^3^
Orthorhombic, *P*2_1_2_1_2_1_	Mo *K*α radiation, λ = 0.71073 Å
Hall symbol: P2ac2ab	Cell parameters from 5140 reflections
*a* = 6.22600 (1) Å	θ = 2.4–24.1°
*b* = 8.3968 (2) Å	µ = 0.07 mm^−^^1^
*c* = 33.5357 (7) Å	*T* = 293 K
*V* = 1753.20 (6) Å^3^	Prism, colourless
*Z* = 4	0.45 × 0.22 × 0.16 mm

### Data collection

**Table:**

Bruker APEXII CCD area-detector diffractometer	2352 independent reflections
Radiation source: fine-focus sealed tube	2014 reflections with *I* > 2σ(*I*)
Graphite monochromator	*R*_int_ = 0.032
φ and ω scans	θ_max_ = 27.5°, θ_min_ = 2.4°
Absorption correction: multi-scan (*APEX2*; Bruker,2005)	*h* = −8→8
*T*_min_ = 0.969, *T*_max_ = 0.989	*k* = −10→9
15454 measured reflections	*l* = −43→43

### Refinement

**Table:**

Refinement on *F*^2^	Primary atom site location: structure-invariant direct methods
Least-squares matrix: full	Secondary atom site location: difference Fourier map
*R*[*F*^2^ > 2σ(*F*^2^)] = 0.035	Hydrogen site location: inferred from neighbouring sites
*wR*(*F*^2^) = 0.091	H-atom parameters constrained
*S* = 1.04	*w* = 1/[σ^2^(*F*_o_^2^) + (0.0481*P*)^2^ + 0.1328*P*] where *P* = (*F*_o_^2^ + 2*F*_c_^2^)/3
2352 reflections	(Δ/σ)_max_ = 0.001
235 parameters	Δρ_max_ = 0.11 e Å^−^^3^
0 restraints	Δρ_min_ = −0.14 e Å^−^^3^

### Special details

**Table:**

Geometry. All e.s.d.'s (except the e.s.d. in the dihedral angle between two l.s. planes) are estimated using the full covariance matrix. The cell e.s.d.'s are taken into account individually in the estimation of e.s.d.'s in distances, angles and torsion angles; correlations between e.s.d.'s in cell parameters are only used when they are defined by crystal symmetry. An approximate (isotropic) treatment of cell e.s.d.'s is used for estimating e.s.d.'s involving l.s. planes.
Refinement. Refinement of *F*^2^ against ALL reflections. The weighted *R*-factor *wR* and goodness of fit *S* are based on *F*^2^, conventional *R*-factors *R* are based on *F*, with *F* set to zero for negative *F*^2^. The threshold expression of *F*^2^ > σ(*F*^2^) is used only for calculating *R*-factors(gt) *etc*. and is not relevant to the choice of reflections for refinement. *R*-factors based on *F*^2^ are statistically about twice as large as those based on *F*, and *R*- factors based on ALL data will be even larger.

### Fractional atomic coordinates and isotropic or equivalent isotropic displacement parameters (Å^2^)

**Table:**

	*x*	*y*	*z*	*U*_iso_*/*U*_eq_	
C1	0.6920 (3)	0.3409 (2)	0.20770 (5)	0.0533 (5)	
H1	0.6063	0.2764	0.1917	0.064*	
C2	0.8720 (4)	0.4144 (2)	0.19215 (5)	0.0578 (5)	
H2	0.9063	0.4002	0.1654	0.069*	
C3	1.0021 (3)	0.5087 (3)	0.21587 (5)	0.0581 (5)	
H3	1.1234	0.5565	0.2050	0.070*	
C4	0.9531 (3)	0.5326 (2)	0.25577 (5)	0.0503 (4)	
H4	1.0414	0.5950	0.2718	0.060*	
C5	0.7212 (3)	0.5705 (2)	0.34358 (4)	0.0424 (4)	
H5	0.8501	0.6264	0.3441	0.051*	
C6	0.5806 (3)	0.5777 (2)	0.37597 (5)	0.0451 (4)	
C7	0.3899 (3)	0.4923 (3)	0.37462 (5)	0.0572 (5)	
H7	0.2957	0.4980	0.3961	0.069*	
C8	0.3361 (3)	0.3984 (3)	0.34195 (5)	0.0599 (5)	
H8	0.2081	0.3413	0.3416	0.072*	
C9	0.4518 (3)	0.3053 (2)	0.27093 (5)	0.0527 (5)	
H9A	0.4584	0.1908	0.2746	0.063*	
H9B	0.3175	0.3326	0.2579	0.063*	
C10	0.6411 (3)	0.36460 (19)	0.24736 (5)	0.0445 (4)	
C11	0.7703 (3)	0.46193 (19)	0.27133 (4)	0.0409 (4)	
C12	0.6660 (3)	0.47883 (19)	0.31054 (4)	0.0394 (4)	
C13	0.4749 (3)	0.3913 (2)	0.31014 (5)	0.0457 (4)	
C1'	0.6708 (4)	0.9656 (3)	0.38487 (6)	0.0685 (6)	
H1'	0.5425	0.9613	0.3707	0.082*	
C2'	0.7935 (6)	1.1033 (3)	0.38502 (7)	0.0870 (9)	
H2'	0.7461	1.1922	0.3710	0.104*	
C3'	0.9840 (6)	1.1101 (3)	0.40558 (7)	0.0895 (9)	
H3'	1.0644	1.2035	0.4051	0.107*	
C4'	1.0581 (4)	0.9809 (3)	0.42685 (6)	0.0748 (7)	
H4'	1.1876	0.9861	0.4406	0.090*	
C5'	1.1220 (4)	0.6453 (3)	0.47611 (6)	0.0678 (6)	
H5'	1.2371	0.7117	0.4821	0.081*	
C6'	1.1042 (5)	0.4983 (3)	0.49389 (6)	0.0810 (7)	
H6'	1.2075	0.4656	0.5122	0.097*	
C7'	0.9355 (5)	0.3993 (3)	0.48493 (6)	0.0841 (8)	
H7'	0.9269	0.2998	0.4970	0.101*	
C8'	0.7779 (4)	0.4454 (3)	0.45821 (5)	0.0665 (6)	
H8'	0.6643	0.3776	0.4522	0.080*	
C9'	0.6353 (3)	0.6749 (2)	0.41279 (5)	0.0484 (4)	
H9'	0.5018	0.6934	0.4276	0.058*	
C10'	0.7422 (3)	0.8349 (2)	0.40610 (5)	0.0514 (5)	
C11'	0.9357 (3)	0.8430 (2)	0.42730 (5)	0.0539 (5)	
C12'	0.9652 (3)	0.6932 (2)	0.44908 (5)	0.0512 (5)	
C13'	0.7921 (3)	0.5934 (2)	0.44068 (4)	0.0486 (4)	

### Atomic displacement parameters (Å^2^)

**Table:**

	*U*^11^	*U*^22^	*U*^33^	*U*^12^	*U*^13^	*U*^23^
C1	0.0685 (12)	0.0461 (10)	0.0453 (9)	0.0044 (10)	−0.0133 (9)	−0.0061 (8)
C2	0.0726 (12)	0.0581 (11)	0.0425 (9)	0.0099 (11)	0.0001 (9)	−0.0040 (8)
C3	0.0576 (11)	0.0652 (12)	0.0515 (10)	0.0027 (11)	0.0064 (8)	−0.0017 (9)
C4	0.0454 (9)	0.0568 (10)	0.0487 (9)	−0.0012 (9)	−0.0007 (7)	−0.0064 (8)
C5	0.0395 (8)	0.0459 (9)	0.0418 (8)	−0.0055 (8)	−0.0037 (7)	−0.0027 (7)
C6	0.0460 (9)	0.0486 (10)	0.0406 (8)	−0.0027 (8)	−0.0032 (7)	0.0011 (7)
C7	0.0518 (10)	0.0723 (13)	0.0474 (9)	−0.0129 (11)	0.0049 (8)	−0.0008 (9)
C8	0.0533 (11)	0.0703 (13)	0.0560 (10)	−0.0224 (11)	−0.0015 (9)	0.0000 (9)
C9	0.0563 (10)	0.0489 (10)	0.0527 (9)	−0.0080 (9)	−0.0127 (9)	−0.0048 (8)
C10	0.0519 (9)	0.0358 (8)	0.0457 (8)	0.0059 (8)	−0.0106 (7)	0.0003 (7)
C11	0.0444 (8)	0.0365 (8)	0.0417 (8)	0.0053 (8)	−0.0071 (7)	−0.0017 (7)
C12	0.0402 (8)	0.0372 (8)	0.0409 (8)	0.0011 (7)	−0.0052 (6)	0.0010 (7)
C13	0.0477 (9)	0.0439 (9)	0.0456 (8)	−0.0082 (9)	−0.0078 (7)	0.0014 (7)
C1'	0.0966 (17)	0.0588 (12)	0.0499 (10)	0.0056 (14)	0.0028 (11)	−0.0061 (9)
C2'	0.148 (3)	0.0539 (13)	0.0587 (13)	−0.0025 (18)	0.0172 (16)	−0.0040 (11)
C3'	0.141 (3)	0.0634 (15)	0.0643 (13)	−0.0384 (18)	0.0314 (17)	−0.0150 (12)
C4'	0.0866 (15)	0.0826 (16)	0.0553 (11)	−0.0337 (15)	0.0164 (11)	−0.0219 (12)
C5'	0.0586 (11)	0.0961 (17)	0.0488 (10)	0.0035 (13)	−0.0035 (9)	−0.0247 (12)
C6'	0.0970 (17)	0.0944 (18)	0.0515 (11)	0.0283 (17)	−0.0225 (12)	−0.0155 (13)
C7'	0.130 (2)	0.0692 (14)	0.0530 (11)	0.0115 (17)	−0.0230 (14)	−0.0030 (11)
C8'	0.0916 (16)	0.0617 (12)	0.0463 (9)	−0.0063 (13)	−0.0088 (11)	−0.0038 (9)
C9'	0.0479 (9)	0.0566 (11)	0.0406 (8)	−0.0019 (9)	0.0039 (7)	−0.0064 (8)
C10'	0.0649 (12)	0.0515 (10)	0.0379 (8)	−0.0024 (10)	0.0085 (8)	−0.0105 (8)
C11'	0.0619 (11)	0.0610 (11)	0.0389 (8)	−0.0110 (10)	0.0108 (8)	−0.0151 (8)
C12'	0.0499 (10)	0.0679 (12)	0.0359 (7)	−0.0042 (10)	0.0060 (7)	−0.0145 (8)
C13'	0.0566 (10)	0.0562 (10)	0.0330 (7)	−0.0004 (9)	0.0022 (7)	−0.0087 (7)

### Geometric parameters (Å, º)

**Table:**

C1—C2	1.382 (3)	C1'—C10'	1.382 (3)
C1—C10	1.382 (2)	C1'—C2'	1.385 (4)
C1—H1	0.9300	C1'—H1'	0.9300
C2—C3	1.384 (3)	C2'—C3'	1.374 (4)
C2—H2	0.9300	C2'—H2'	0.9300
C3—C4	1.387 (2)	C3'—C4'	1.378 (4)
C3—H3	0.9300	C3'—H3'	0.9300
C4—C11	1.386 (2)	C4'—C11'	1.387 (3)
C4—H4	0.9300	C4'—H4'	0.9300
C5—C12	1.392 (2)	C5'—C6'	1.375 (4)
C5—C6	1.396 (2)	C5'—C12'	1.392 (3)
C5—H5	0.9300	C5'—H5'	0.9300
C6—C7	1.388 (3)	C6'—C7'	1.373 (4)
C6—C9'	1.519 (2)	C6'—H6'	0.9300
C7—C8	1.391 (3)	C7'—C8'	1.384 (3)
C7—H7	0.9300	C7'—H7'	0.9300
C8—C13	1.374 (3)	C8'—C13'	1.378 (3)
C8—H8	0.9300	C8'—H8'	0.9300
C9—C10	1.504 (2)	C9'—C13'	1.515 (2)
C9—C13	1.507 (2)	C9'—C10'	1.516 (3)
C9—H9A	0.9700	C9'—H9'	0.9800
C9—H9B	0.9700	C10'—C11'	1.401 (3)
C10—C11	1.400 (2)	C11'—C12'	1.466 (3)
C11—C12	1.473 (2)	C12'—C13'	1.394 (3)
C12—C13	1.399 (2)		
			
C2—C1—C10	119.00 (17)	C10'—C1'—C2'	118.9 (2)
C2—C1—H1	120.5	C10'—C1'—H1'	120.5
C10—C1—H1	120.5	C2'—C1'—H1'	120.5
C1—C2—C3	120.87 (17)	C3'—C2'—C1'	120.9 (3)
C1—C2—H2	119.6	C3'—C2'—H2'	119.6
C3—C2—H2	119.6	C1'—C2'—H2'	119.6
C2—C3—C4	120.59 (18)	C2'—C3'—C4'	121.1 (2)
C2—C3—H3	119.7	C2'—C3'—H3'	119.5
C4—C3—H3	119.7	C4'—C3'—H3'	119.5
C11—C4—C3	118.81 (18)	C3'—C4'—C11'	118.6 (2)
C11—C4—H4	120.6	C3'—C4'—H4'	120.7
C3—C4—H4	120.6	C11'—C4'—H4'	120.7
C12—C5—C6	119.22 (15)	C6'—C5'—C12'	119.1 (2)
C12—C5—H5	120.4	C6'—C5'—H5'	120.5
C6—C5—H5	120.4	C12'—C5'—H5'	120.5
C7—C6—C5	119.27 (15)	C7'—C6'—C5'	120.7 (2)
C7—C6—C9'	119.73 (16)	C7'—C6'—H6'	119.7
C5—C6—C9'	120.99 (15)	C5'—C6'—H6'	119.7
C6—C7—C8	121.63 (17)	C6'—C7'—C8'	121.0 (2)
C6—C7—H7	119.2	C6'—C7'—H7'	119.5
C8—C7—H7	119.2	C8'—C7'—H7'	119.5
C13—C8—C7	119.00 (17)	C13'—C8'—C7'	118.9 (2)
C13—C8—H8	120.5	C13'—C8'—H8'	120.6
C7—C8—H8	120.5	C7'—C8'—H8'	120.6
C10—C9—C13	103.00 (14)	C13'—C9'—C10'	102.05 (15)
C10—C9—H9A	111.2	C13'—C9'—C6	113.82 (15)
C13—C9—H9A	111.2	C10'—C9'—C6	117.03 (14)
C10—C9—H9B	111.2	C13'—C9'—H9'	107.8
C13—C9—H9B	111.2	C10'—C9'—H9'	107.8
H9A—C9—H9B	109.1	C6—C9'—H9'	107.8
C1—C10—C11	120.33 (17)	C1'—C10'—C11'	120.0 (2)
C1—C10—C9	129.63 (17)	C1'—C10'—C9'	129.7 (2)
C11—C10—C9	109.98 (14)	C11'—C10'—C9'	110.21 (17)
C4—C11—C10	120.37 (15)	C4'—C11'—C10'	120.5 (2)
C4—C11—C12	131.06 (15)	C4'—C11'—C12'	130.8 (2)
C10—C11—C12	108.38 (15)	C10'—C11'—C12'	108.59 (17)
C5—C12—C13	120.54 (15)	C5'—C12'—C13'	120.0 (2)
C5—C12—C11	130.90 (15)	C5'—C12'—C11'	131.35 (19)
C13—C12—C11	108.41 (14)	C13'—C12'—C11'	108.56 (16)
C8—C13—C12	120.32 (16)	C8'—C13'—C12'	120.38 (19)
C8—C13—C9	129.64 (16)	C8'—C13'—C9'	128.99 (19)
C12—C13—C9	109.97 (15)	C12'—C13'—C9'	110.59 (16)
			
C10—C1—C2—C3	0.9 (3)	C12'—C5'—C6'—C7'	0.6 (3)
C1—C2—C3—C4	−0.6 (3)	C5'—C6'—C7'—C8'	−0.8 (4)
C2—C3—C4—C11	−0.7 (3)	C6'—C7'—C8'—C13'	−0.2 (3)
C12—C5—C6—C7	0.5 (3)	C7—C6—C9'—C13'	100.0 (2)
C12—C5—C6—C9'	179.39 (16)	C5—C6—C9'—C13'	−78.9 (2)
C5—C6—C7—C8	0.7 (3)	C7—C6—C9'—C10'	−141.21 (18)
C9'—C6—C7—C8	−178.28 (19)	C5—C6—C9'—C10'	39.9 (2)
C6—C7—C8—C13	−0.5 (3)	C2'—C1'—C10'—C11'	0.0 (3)
C2—C1—C10—C11	0.1 (3)	C2'—C1'—C10'—C9'	176.29 (18)
C2—C1—C10—C9	177.08 (18)	C13'—C9'—C10'—C1'	−176.25 (18)
C13—C9—C10—C1	−172.24 (18)	C6—C9'—C10'—C1'	58.8 (3)
C13—C9—C10—C11	4.98 (19)	C13'—C9'—C10'—C11'	0.32 (17)
C3—C4—C11—C10	1.7 (3)	C6—C9'—C10'—C11'	−124.61 (17)
C3—C4—C11—C12	−172.68 (18)	C3'—C4'—C11'—C10'	0.8 (3)
C1—C10—C11—C4	−1.4 (2)	C3'—C4'—C11'—C12'	−175.29 (19)
C9—C10—C11—C4	−178.95 (15)	C1'—C10'—C11'—C4'	−0.7 (3)
C1—C10—C11—C12	174.13 (15)	C9'—C10'—C11'—C4'	−177.67 (16)
C9—C10—C11—C12	−3.39 (18)	C1'—C10'—C11'—C12'	176.20 (17)
C6—C5—C12—C13	−1.7 (2)	C9'—C10'—C11'—C12'	−0.76 (18)
C6—C5—C12—C11	173.23 (16)	C6'—C5'—C12'—C13'	0.6 (3)
C4—C11—C12—C5	−0.3 (3)	C6'—C5'—C12'—C11'	176.7 (2)
C10—C11—C12—C5	−175.22 (17)	C4'—C11'—C12'—C5'	1.0 (3)
C4—C11—C12—C13	175.13 (17)	C10'—C11'—C12'—C5'	−175.45 (17)
C10—C11—C12—C13	0.20 (18)	C4'—C11'—C12'—C13'	177.41 (18)
C7—C8—C13—C12	−0.8 (3)	C10'—C11'—C12'—C13'	0.92 (18)
C7—C8—C13—C9	−177.28 (19)	C7'—C8'—C13'—C12'	1.5 (3)
C5—C12—C13—C8	1.9 (2)	C7'—C8'—C13'—C9'	−175.90 (19)
C11—C12—C13—C8	−174.10 (17)	C5'—C12'—C13'—C8'	−1.7 (3)
C5—C12—C13—C9	179.04 (16)	C11'—C12'—C13'—C8'	−178.54 (17)
C11—C12—C13—C9	3.06 (18)	C5'—C12'—C13'—C9'	176.14 (15)
C10—C9—C13—C8	171.96 (19)	C11'—C12'—C13'—C9'	−0.72 (18)
C10—C9—C13—C12	−4.86 (19)	C10'—C9'—C13'—C8'	177.84 (18)
C10'—C1'—C2'—C3'	0.6 (3)	C6—C9'—C13'—C8'	−55.1 (2)
C1'—C2'—C3'—C4'	−0.4 (4)	C10'—C9'—C13'—C12'	0.26 (17)
C2'—C3'—C4'—C11'	−0.3 (3)	C6—C9'—C13'—C12'	127.29 (16)
